# Invasive Lobular Carcinoma with Extracellular Mucin Production: Description of a Case and Review of the Literature

**DOI:** 10.7759/cureus.5550

**Published:** 2019-09-01

**Authors:** Nektarios Koufopoulos, Foteini Antoniadou, Stefania Kokkali, Eleni Pigadioti, Lubna Khaldi

**Affiliations:** 1 Pathology, Attikon University Hospital, Medical School of Athens, Athens, GRC; 2 Pathology, Saint Savvas Cancer Hospital, Athens, GRC; 3 Oncology, Saint Savvas Cancer Hospital, Athens, GRC; 4 Pathology, Metropolitan Hospital of Athens, Athens, GRC

**Keywords:** invasive lobular breast cancer, extracellular mucin production, signet ring, mucinous carcinoma of the breast, e-cadherin

## Abstract

Invasive lobular carcinoma of the breast is the second most common subtype of breast carcinoma. It accounts for 5-15% of the breast carcinoma cases reported. It shows a different metastatic pattern compared to invasive carcinoma of no special type. There are several variants of this cancer including the classic, solid, signet ring cell, tubulolobular, alveolar, trabecular, pleomorphic, and mixed subtypes each one with a distinctive morphology. Invasive lobular carcinoma has been associated with intracellular mucin production, in the form of intracytoplasmic lumina and signet ring cells whereas extracellular mucin production has been related to carcinomas of ductal origin. A new variant that displays extracellular mucin production was described recently. Only nineteen cases of this rare entity have been reported to date. In this manuscript, we report an additional case of invasive lobular carcinoma with extracellular mucin production, describing its clinico-pathological characteristics, and review the literature on the same.

## Introduction

Invasive lobular carcinoma (ILC) of the breast is the second most common variant of breast carcinoma. It accounts for 5-15% of invasive mammary carcinomas [1]. It usually affects older patients. It is more often bilateral and multifocal or multicentric compared to invasive carcinoma of no special type. Also, the metastatic pattern of ILC is different, showing a tendency to metastasize to the gastrointestinal tract, meninges, bone and the female genital tract [1].

Histologically, the classic variant of ILC is characterized by distinct morphology that consists of small cells lacking cohesion arranged in a single file pattern, and sometimes in a concentric pattern around existing ducts and lobular units [2]. Tumor cells have intra-cytoplasmic lumina, and a variable number of signet ring cells (SRC) are present. The mitotic count is low. Several different histological variants have been described including the solid, signet ring cell, tubulolobular, alveolar, trabecular, pleomorphic, and mixed variants. Stromal mucin is present in several benign or malignant breast lesions. Traditionally extracellular mucin production is associated with carcinomas of ductal origin. Furthermore, some authors consider mucinous cystadenocarcinoma of the breast to originate from mucinous metaplasia [3].

In 2009 Rosa et al. [4] described a new variant of ILC that displayed extracellular mucin production after which 20 cases have been reported in the English literature [2, 5-11]. In this manuscript, we report an additional case of ILC with extracellular mucin production. We also review the literature and discuss the differential diagnosis.

## Case presentation

**Figure 1:**
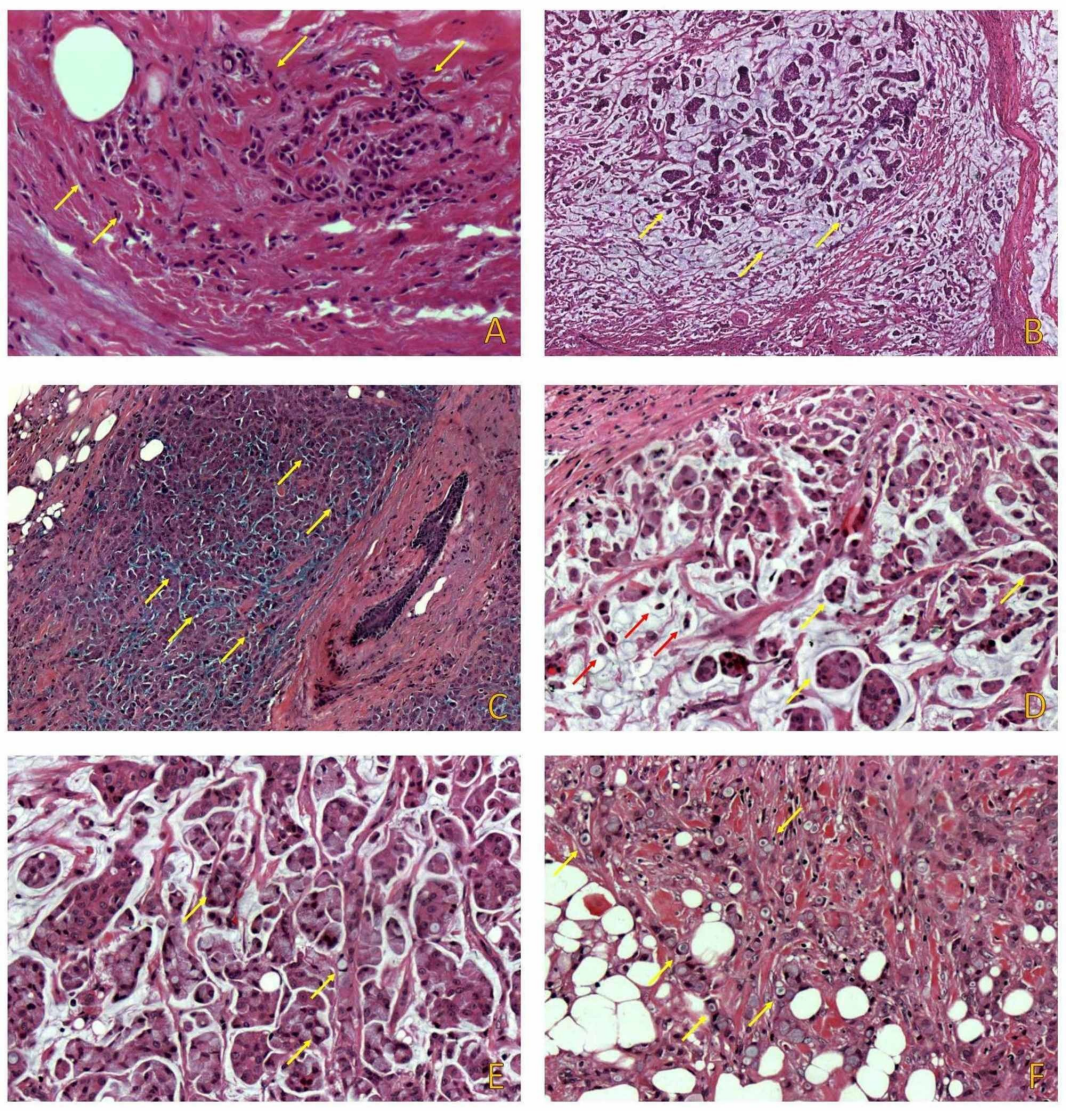
The tumor consisted of two distinct components. The first component consisted of ILC of the classical subtype (A) and the second one of a mucinous component consisting of multiple, circumscribed, nodules (B), and patchy extracellular mucin with irregular borders (C). In the mucinous area (D) single tumor cells (red arrows), as well as clusters (yellow arrows) were identified. SRCs were present in both areas (E, F). ILC: Invasive lobular carcinoma SRCs: Signet ring cells

A 65-year-old female patient was admitted to our hospital's surgical department after discovering a small palpable lump on the left upper quadrant of her right breast during self-examination. Upon physical examination, a solid mass with a maximum diameter of around 2cm with an irregular contour was detected upon palpation. Imaging studies were consistent with malignancy. During surgery, frozen sections of the tumor and sentinel lymph node biopsy were performed. The breast lump was positive for malignancy. Sentinel lymph node was negative for metastatic disease. A simple right mastectomy was performed. On gross examination, the tumor was soft, pale, grayish blue, gelatin-like, and well-circumscribed. On microscopic examination, the tumor consisted of two separate foci measuring 13 and 6mm, respectively. Both foci consisted of a non-mucinous ILC component of the classical (figure 1A) and solid variant and a mucinous component. The mucinous component represented about 20% of the tumor. Extracellular mucin production was seen in the form of multiple, relatively circumscribed, nodular areas (figure 1B), and patchy extracellular mucin production with irregular borders (figure 1C). Tumor cells in the mucinous component were arranged in clusters (figure 1D) and single cells. Several signet ring cells were identified in both areas (figure 1E, 1F).

**Figure 2:**
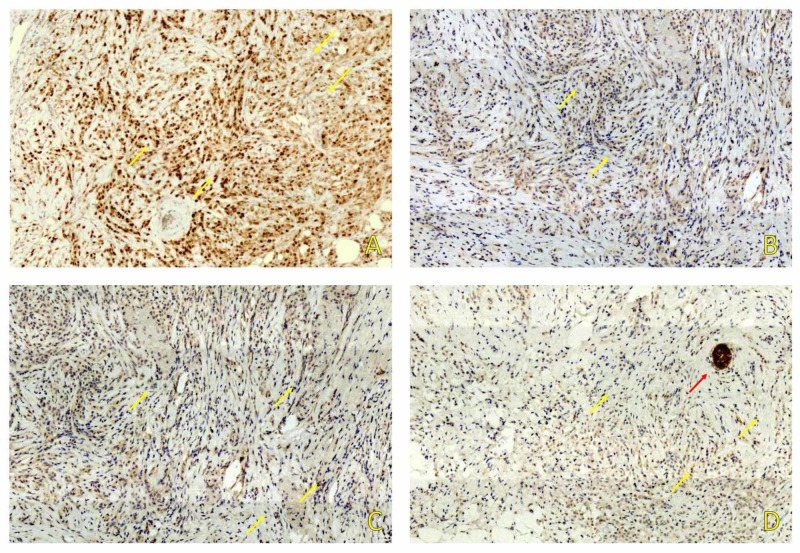
Immunohistochemical study showed positive staining for ER (A) and negative for PR (B) and HER-2/neu (C). E-Cadherin (D) was negative in tumor cells (yellow arrows) and positive in normal ductal structures (red arrow). ER: Estrogen receptors PR: Progesterone receptors

Using Immunohistochemistry, the tumor cells stained positive for estrogen receptors (ER) (figure 2A) and negative for progesterone receptors (PR) (figure 2B), HER-2 (figure 2C), and E-Cadherin (figure 2D). Proliferation index Ki-67 stained 10% of tumor nuclei. Based on morphological and immunohistochemistry results, the tumor was signed out as ILC Grade 2 with extracellular mucin production.

A multidisciplinary tumor board suggested that administration of adjuvant chemotherapy was not appropriate. Thus, radiotherapy and hormonal therapy were offered. Ten months after surgery, the patient is alive without evidence of recurrence or metastasis.

## Discussion

ILC with extracellular mucin production is a rare variant of ILC. Our literature review revealed seven articles describing single cases and two case series describing eight and four cases, respectively [2,4-11]. Several articles do not report relevant data, such as lymph node status and treatment. Information concerning follow-up and outcome were published only in one case series [9].

All patients were female. Patients’ age ranged from 38 to 87 years (median age 64). Most patients presented with a palpable mass and/or a mammographic abnormality. Tumor median size was 43 mm (range 8-100 mm). All previous cases comprised two components, a mucinous and a non-mucinous one. The mucinous part consisted either of nodular lakes of mucin or was patchy with irregular borders and represented 10 to 70% of the tumor area. Seven patients presented with a grade 2 tumor, five of which had a grade 3 tumor and only two patients had a grade 1 tumor. In all tumors except for one case in the series reported by Singh et al., variable numbers of SRC were found [10]. Rarely, E-cadherin negative glandular or pseudocribriform formations were observed. Nine out of sixteen cases with available information had lymph node metastasis. In twelve out of nineteen cases, lobular neoplasia was present.

In all cases with available data, ER was positive, PR was positive in ten and negative in four cases, and Her-2 was over-expressed in two, negative in eleven, and was scored 1+ in one and 2+ in two cases. None of these cases were amplified with fluorescent in situ hybridization. Ki67 proliferation index expression ranged from 7 to 40%. The clinico-pathological features of all previous cases are presented in Table 1.

**Table 1:** ILC with extracellular mucin production clinicopathological data. Abbreviations: mm; millimeter, NA; not available P; positive, N; negative, Namp; not amplified, ER; estrogen receptor, PR; progesterone receptor, *; no information, **; isolated tumor cells were found in one lymph node ILC; invasive lobular carcinoma

Case	Author	Year	Age	Size (mm)	LΝ	Grade	ER %	PR %	HER2	Ki-67 (%)
1	Rosa et al. [4]	2009	60	90	NA	NA	NA	NA	NA	NA
2	Yu et al. [2]	2010	65	ΝΑ	1+ *	NA	100	N	3+	25
3	Haltas et al. [5]	2012	43	ΝΑ	1/19	NA	P	P	N	NA
4	Bari et al. [6]	2015	38	35	2/10	NA	P	P	N	NA
5	Gomez-Macias et al. [7]	2016	60	9	0/4	1	P	90	N	NA
6	Cserni et al. [9]	2017	69	>24	1/2	2	100	10	N	20
7	Cserni et al. [9]	2017	65	90	11/13	2	90	5	N	30
8	Cserni et al. [9]	2017	71	46	0/3	2	90	40	3+	10
9	Cserni et al. [9]	2017	62	80	10/23	2	90	80	Namp	40
10	Cserni et al. [9]	2017	45	29	0/2	3	95	95	N	40
11	Cserni et al. [9]	2017	56	22	0/1	2	100	70	N	20
12	Cserni et al. [9]	2017	75	30	7/9	2/3	80	<1	N	20
13	Cserni et al. [9]	2017	60	50	3/13	2	60	5	Namp	7
14	Boukhechba et al. [8]	2018	75	15	NA	NA	P	N	N	NA
15	Singh et al. [10]	2019	87	100	1/3	2	P	NA	N	NA
16	Singh et al. [10]	2019	72	16	NA	3	P	NA	N	NA
17	Singh et al. [10]	2019	70	>20	0/10	3	P	NA	N	NA
18	Singh et al. [10]	2019	77	8	0/2	1	P	NA	N	NA
19	Baig et al. [11]	2019	67	60	0/2**	3	P	N	Namp	NA
20	Present case	2019	65	13	0/2	2	100	N	N	10

All patients underwent surgical treatment. Six patients were treated with breast-conserving surgery (BCS), five of which underwent a mastectomy and one of them was treated with excisional biopsy (EB). Three patients, two treated initially with BCS, and one with EB underwent a mastectomy subsequently. Sentinel lymph node biopsy was performed in six, and axillary lymph node dissection in five patients. Lymph node metastasis was found in nine out of sixteen patients with reported nodal status. Hormonal therapy, chemotherapy, and radiotherapy were administered in five, four, and eight patients, respectively. One patient was treated initially with neoadjuvant therapy. Treatment features are reported in table 2.

**Table 2:** ILC with extracellular mucin production therapeutic approach features. Abbreviations: M; mastectomy, NA; not available, BCS; breast-conserving surgery, SLNB;  sentinel lymph node biopsy, ALND; axillary lymph node dissection, RT; radiotherapy, HT; hormonal therapy, ChT; chemotherapy, Neoadj; neoadjuvant, EB; Excisional biopsy, ILC; invasive lobular carcinoma

Case	Author	Surgery	Adjuvant therapy	Follow up (months)
1	Rosa et al. [4]	M	NA	NA
2	Yu et al. [2]	BCS + SLNB	NA	NA
3	Haltas et al. [5]	M + ALND	NA	NA
4	Bari et al. [6]	M + ALND	NA	NA
5	Gomez-Macias et al. [7]	BCS + SLNB	RT + HT	NA
6	Cserni et al. [9]	M + SLNB	RT + HT	26
7	Cserni et al. [9]	BCS > M	RT + HT + ChT	40
8	Cserni et al. [9]	BCS + SNB	RT + HT + ChT	29
9	Cserni et al. [9]	BCS > M + ALND	RT	68
10	Cserni et al. [9]	BCS + SLNB	RT + ChT	2
11	Cserni et al. [9]	M + SLNB	RT	11
12	Cserni et al. [9]	EB > M + ALND	Neoadj ChT	21
13	Cserni et al. [9]	M + ALND	RT + HT + ChT	NA
14	Boukhechba et al. [8]	NA	NA	NA
15	Singh et al. [10]	NA	NA	NA
16	Singh et al. [10]	NA	NA	NA
17	Singh et al. [10]	NA	NA	NA
18	Singh et al. [10]	NA	NA	NA
19	Baig et al. [11]	BCS	ChT+RT+HT	NA
20	Present case	M+ SLNB	RT + HT	8

Some of the previously reported cases were initially misdiagnosed as mucinous carcinomas or as invasive carcinomas of no special type with extracellular mucin production. This may be explained by the fact that the initial diagnosis was made in limited biopsy specimens in which only the mucinous area was sampled [11]. The differential diagnosis includes pure mucinous carcinoma, mixed mucinous- no special type carcinoma, solid papillary carcinoma, polymorphous mammary carcinoma [9], and metaplastic matrix-producing carcinoma [12]. Pure mucinous carcinoma lacks the component with the morphological characteristics of ILC. Mixed mucinous-no special type carcinoma or mixed mucinous-ILC may be more diagnostically challenging since two different components are present that may sometimes simulate those found in ILC with extracellular mucin production. Careful examination with attention to morphology will distinguish most if not all cases. Solid papillary carcinoma may occasionally be associated with extracellular mucin production and invasive mucinous carcinoma [9], but it lacks the distinctive morphology of ILC. The mucoid-like stroma of polymorphous mammary adenocarcinoma and matrix-producing carcinoma may be confused with the mucin lakes of ILC with extracellular mucin production. Lack of expression of estrogen and progesterone receptors in both tumors as well as other characteristics will help distinguish them from ILC [12,13]. In all the previously mentioned entities, when facing diagnostic difficulty, an immunohistochemical panel consisting of E-cadherin, b-catenin, and P120 immunostains will give the solution. We believe that the precise diagnosis of this variant of ILC is essential since several of its histological mimics have a different treatment and prognosis.

## Conclusions

ILC with extracellular mucin production is a rare variant of ILC with a distinct morphology. Its diagnosis may occasionally be challenging, especially when dealing with limited biopsy specimens. The diagnosis of a mucinous carcinoma should not be made just by examining a tumor on low power. The presence of discohesive growth, bland cytological features, intracytoplasmic lumina, and SRC found on high power examination, should alert the pathologist. The use of appropriate immunohistochemical stains will assist in avoiding a misdiagnosis. Precise diagnosis is the key to correct treatment since most of its histological mimics have different treatment and prognosis.
